# Lattice bond chains administer kink anisotropy and inform strategies of theophylline crystal self-healing

**DOI:** 10.1016/j.isci.2025.112866

**Published:** 2025-06-10

**Authors:** Angelica Niazov-Elkan, Huan-Jui Lee, Sima Mafi, Manasa Yerragunta, Irit Rosenhek-Goldian, Marcos Penedo, Georg Fantner, Anna Kossoy, Yishay Feldman, Yael Diskin-Posner, Dan Oron, Peter G. Vekilov

**Affiliations:** 1 Department of Molecular Chemistry and Materials Science, Weizmann Institute of Science, Rehovot 7610001, Israel; 2 William A. Brookshire Department of Chemical and Biomolecular Engineering, University of Houston, 4226 Martin Luther King Boulevard, Houston, TX 77204-4004, USA; 3 Welch Center for Advanced Bioactive Materials Crystallization, University of Houston, 4226 M.L. King Boulevard, Houston, TX 77204-4004, USA; 4 Department of Chemical Research Support Weizmann Institute of Science Rehovot, Rehovot 7610001, Israel; 5 École Polytechnique Fédérale de Lausanne, Laboratory for Bio- and Nano-Instrumentation, CH1015 Lausanne, Switzerland; 6 Department of Chemistry, University of Houston, 3585 Cullen Boulevard, Houston, TX 77204-5003, USA

**Keywords:** Chemistry, Physics, Materials science

## Abstract

How molecular-level understanding of the crystal growth mechanisms and their relation to lattice bonds informs the rational design of crystals with desired shapes and properties has remained elusive. Here we employ theophylline crystals and drive them into classical growth mode, in which the crystals grow molecule-by-molecule and new layers are generated by two-dimensional nucleation. We demonstrate that classical growth allows for controlling the crystal’s shape and dimensions. We correlate the anisotropic responses to the supersaturation of the growth rates of crystal layers and crystal faces to the hydrogen and π−π stacking bond chains in the crystal lattice. The obtained insights suggest strategies to direct the crystal shape to either one-dimensional needles or flat sheets. Moreover, we show that crystals that grow by the classical mode of direct monomer incorporation have the potential to regrow and heal once a defect is introduced by mechanical cut or local thermal subliming of crystalline sections.

## Highlights

• The strengths of the lattice bonds control the growth anisotropy
• Crystal growth in the classical regime grants the means to control crystal morphology
• Transition between needle and slate crystal shape can be driven by supersaturation
• Crystals that grow classically regenerate after the introduction of cuts

## Graphical abstract

**Figure:**
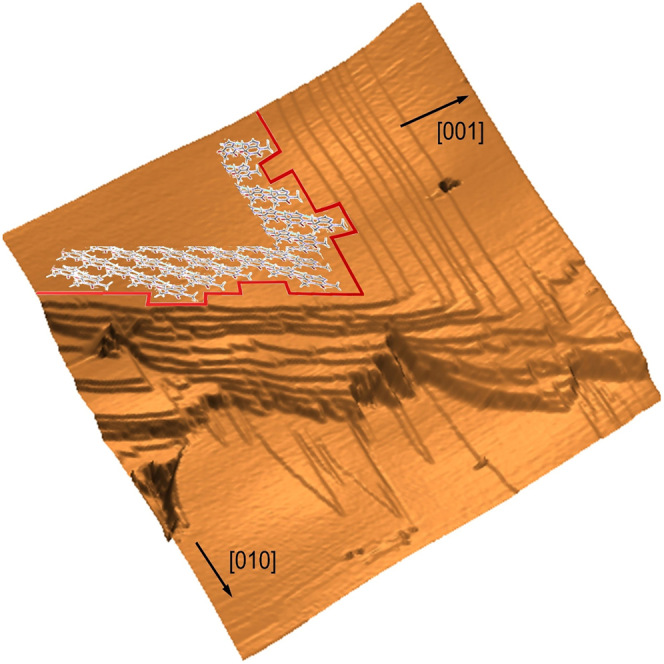

## Introduction

Solution-grown crystals play important roles in fundamental and applied fields.^1^^,^^2^^,^^3^^,^^4^^,^^5^ Even though growing crystals from solution is the most facile and scalable method, the ability to design crystals with desired properties is still limited.^1^ The need for predictive control of crystals' physical and chemical properties has driven research efforts to elucidate the fundamental processes of crystallization and employ the obtained insights to design sophisticated methods to prepare new and improved crystalline materials.^4^^,^^5^

Thin organic crystals that exhibit large optical and structural anisotropy hold significant potential as platforms for polarization-sensitive light manipulating materials and piezoelectric and dielectric devices. For instance, we previously reported the development of a synthetic method to obtain a highly oriented birefringent crystalline macro-surface constructed of small organic molecules of the xanthine family, which may find application as metasurfaces for thin polarization holography.^6^

Crystals grow from solutions by classical or nonclassical mechanisms. In the classical pathway, nucleation and growth occur via molecule-by-molecule attachment to create the final crystalline structure.^7^^,^^8^^,^^9^^,^^10^ Recently, nonclassical pathways have been discovered that involve a diverse set of precursors that range in complexity from oligomeric species and primary particles to bulk amorphous phases and small crystallites.^11^^,^^12^^,^^13^^,^^14^^,^^15^^,^^16^ Nonclassical crystal growth can involve a dynamic sequence of events that include precursor attachment and structural rearrangement^11^^,^^17^^,^^18^^,^^19^^,^^20^^,^^21^^,^^22^, leading to the formation of crystals that often exhibit a markedly different habit than those formed via classical pathways.^12^ It was previously shown that for crystal growing from purely organic solvents, solute molecules reach the growth sites on the steps, the kinks directly from the solution.^23^^,^^24^ The preference for the direct incorporation pathway in organic solvents was attributed to the strong adsorption of solute to the surface of organic crystals, which hampers diffusion along the surface.^23^^,^^24^ By contrast, during crystallization from solvents that contain any amounts of water, where the solute-crystal surface bond is weakened by the 3D hydrogen bonded network of water, a kink access pathway that includes the adsorption of the solvent on the crystal surface, followed by two-dimensional diffusion toward the steps, appears to be favored. Further studies of the classical crystallization pathways in solution have provided extensive insights into processes at all relevant length scales.^24^^,^^25^^,^^26^^,^^27^^,^^28^^,^^29^ These insights have allowed control over the development of growth instabilities and defects, as well as guiding their morphology and shape,^30^^,^^31^^,^^32^^,^^33^^,^^34^ which can significantly affect the crystal properties.

In this work, we explore the growth mechanism of anhydrous theophylline crystals in an organic solvent. We then employ the obtained growth mode information to control the morphology of the crystals. Theophylline crystals have high optical anisotropy and biaxial birefringence that reaches up to 15% in-plane.^6^ Furthermore, anhydrous theophylline packs into non-centrosymmetric polar crystals with high structural anisotropy. These factors, together with the facile solution fabrication, make the crystals of anhydrous theophylline favored candidates for the manufacture of polarization-sensitive optics and piezoelectric crystalline devices. For these applications, the preferred morphology is flat single-orientation 2D sheets with maximal anisotropy in-plane. Theophylline, however, often forms polymorphs with needle-shaped morphology.

To design theophylline crystals with desired morphology and perfection, we take the insights of the classical mode kinetics one step further and show that following the growth kinetics grants us the possibility to control the shape and the dimensions of the crystal. Moreover, we demonstrate that a crystal that grows by the classical mode of direct monomer incorporation can regrow and heal once a “defect” is introduced by a mechanical cut, or by local thermal subliming of crystalline sections. This would be difficult to achieve in systems that involve growth by the incorporation of clusters or nanoparticles.

Importantly, this growth mode sheds light on the regrowth and regeneration properties of the crystals. We guide the crystals to grow either into needle such as ribbons, or 2D sheets by altering the growth conditions and specifically the degree of supersaturation. We show that the possibility of the regrowth and regeneration of a cleaved crystal depends on the growth kinetics in the relevant direction, and while the cut along a fast-growing direction shows full recovery, the crystal that was cut along the slow-growing direction shows slower regrowth and permanent "scarring."

## Results

### Theophylline crystals growth: Phenomenology

**Figure 1:**
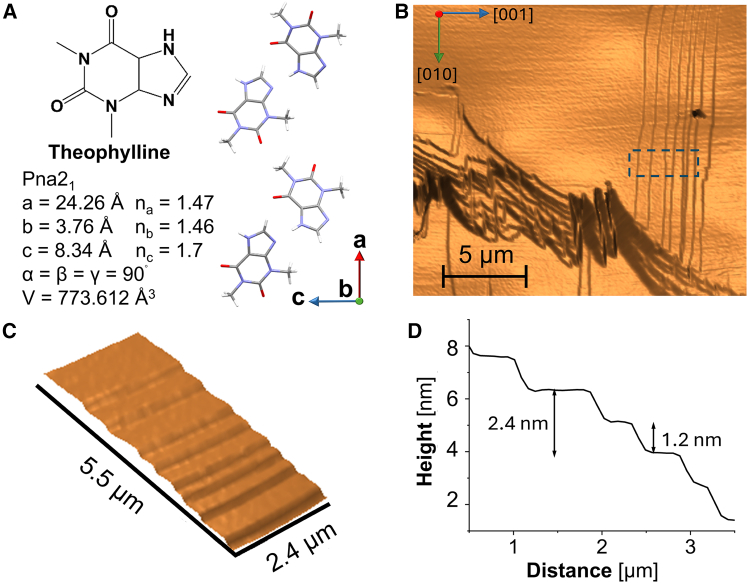
Theophylline crystals and their growth (A) The structure of theophylline and of the anhydrous Form II crystals (Crystallographic symmetry group Pna2_1_, Database: CDC REFCODE: BAPLOT01). C atoms are shown in gray; H, in silver; N, in magenta; and O, in red. The unit cell parameters are listed. (B–D) The morphology of a (200) theophylline crystal surface. (B) An AFM micrograph of the (200) surface in contact with a 0.167 M solution in n-octanol. (C) Expanded view of the area marked with a dashed rectangle in (B). (D) Height profile in direction perpendicular to steps along the center of the image in (C), show the sub-steps with height half a unit cell parameter in the [100] direction.

The common anhydrous form of theophylline are polar orthorhombic crystals with Pna2_1_ symmetry, referred to as Form II (Figure 1A).^35^^,^^36^ These crystals transform into a monohydrate upon exposure to water.^37^^,^^38^ The water solubility in the chosen crystallization solvent, n-octanol, is low, 2.70 mol kg^−1^ ref. ^39^, preventing monohydrate from forming. Thus, polymorphism is eliminated, and theophylline is constrained to the orthorhombic form.^40^ Large, millimeter-range crystals of this form can be grown from n-octanol. We deposit the crystals on a glass substrate and monitor their growth by *in situ* atomic force microscopy (AFM) in supersaturated theophylline solutions. This method provides time-resolved images with high spatial and temporal resolution.

The AFM monitoring demonstrates a strictly classical mechanism, whereby stacks of unfinished layers, whose edges represent the steps, spread along the studied (200) surface (Figure 1B). Dedicated efforts failed to find dislocations outcropping on the (200) face that may be the source of spiral steps. Further observations found that the steps originate at the edges of the crystal (Figure S1), where they are generated by two-dimensional (2D) nucleation; this localization is likely enforced by the higher supersaturation at the crystal’s edges.^41^^,^^42^^,^^43^ Regions of high step density coexist with wide step-free terraces. We attribute the variable step density to step generation by 2D nucleation, which sensitively responds to even minor variations of the supersaturation near the crystal edges, the locations of step generation. Thus, a burst of supersaturation generates a packet of steps with high density, whereas a subsequent supersaturation drop would lead to long step separations. Some of the steps are 2.4 nm high, corresponding to the unit cell parameter |***a***| = 2.413nm (Figures 1B–1D and S2). The terraces between the steps represent the (200) crystal plane. Select steps split into sub-steps of height ca. 1.2 nm, corresponding to half a unit cell parameter in the ***a***, or [100], direction (Figures 1C, 1D, and S3). The two-halves of the unit cell are symmetry-related by a (010) glide plane; thus, the sub-steps grow at the same velocity.

### The molecular mechanism of step growth

**Figure 2:**
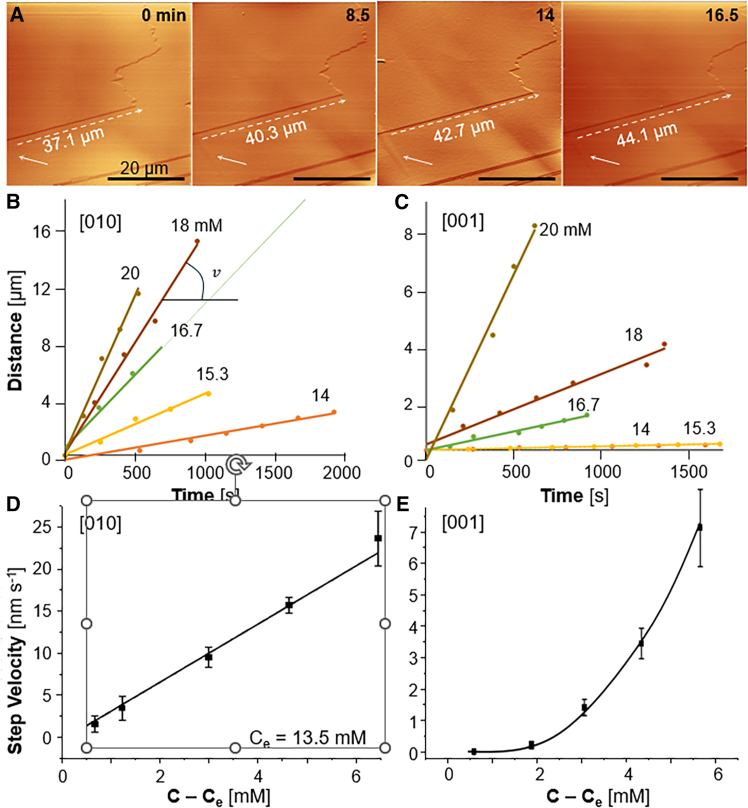
The growth of steps on the (200) crystal face (A) *In situ* AFM monitoring of the displacement of a step growing in the [010] direction in a 16.7 mM theophylline solution in n-octanol at the times indicated in the images. White arrows mark the reference point. (B and C) The evolutions of the step position in time for steps growing in the [010] direction, in (B), and in the [001] direction, in (C), at the theophylline concentrations shown next to each correlation. The step velocity v is evaluated as the slope of each correlation. (D and E) Step velocity v in the [010] direction (D), and [001] direction (E) on (200) as a function of theophylline concentration; error bars represent the standard deviations of the respective slopes in (b) and (c). AFM measurements were performed at a temperature T = 25.2 ± 0.1°C, at which the solubility $C_{e}$ = 13.5 mM.

To deduce how solute molecules that reach the steps incorporate into them, we rely on the correlation of the step velocity v with the solute concentration. The step velocity v is controlled by the rate of the association of molecules to the kinks, the kink density, and the supply of solute molecules to the growing steps. The velocity v was measured by time-resolved *in situ* AFM (Figure 2A), where step displacements along the [001] and [010] directions were tracked with respect to a reference point (Figures 2A–2C) and the average step velocities were evaluated from the slopes of the displacement evolutions (Figures 2B, 2C and S4).

The AFM measurements reveal that the step velocity v in both [010] and [001] directions is steady in time at all tested theophylline concentrations (Figures 2B and 2C). In the [010] direction, v increases linearly with theophylline concentration (Figure 2D) as expected from the classical models of crystal growth^9^^,^^44^^,^^45^ and observed for numerous inorganic, organic, biomineral, and protein crystals^15^^,^^24^^,^^27^^,^^46^^,^^47^^,^^48^^,^^49^^,^^50^^,^^51^^,^^52^^,^^53^^,^^54^^,^^55^^,^^56^^,^^57^^,^^58^^,^^59^^,^^60^ By contrast, v(C) in the [001] direction is superlinear and slower by ca. 20× at low supersaturations and ca. 3× at higher supersaturations (Figures 2D and 2E). The distinct v(C) correlations in the two step directions enforce a rarely observed supersaturation dependent anisotropy of the surface features on the (200) theophylline face.

Time-resolved AFM imaging also reveals that the step velocity is independent of the step density, the step height, and the inter-step distance l, as the steps with different height propagate at the same velocity in both [001] and [010] directions (Figures S5 and S6). These findings indicate that the monomers incorporate into the crystalline steps directly from the solution, rather than adsorbing on terraces between steps and diffusing along the surface to incorporate into the growth sites.^23^ Indeed, if the solute reaches the steps via the crystal surface, the step supply field is constrained to two dimensions, which stunts the growth of closely spaced steps. Concurrently, analytical models of step growth mediated by surface diffusion predict that v scales with $h^{-1}$ and sharply slows down at short *l*.^23^^,^^61^ By contrast, if the steps feed directly from the solution, the supply field is three-dimensional and abundant for closely spaced steps. Closed-form expressions for this growth mode predict negligible v(l) correlations.^23^^,^^44^^,^^62^ The finding that the solute molecules directly reach the steps eliminates possible intermediate stages in the growth mechanism and simplifies the analysis of the step kinetics.

To understand the two distinct v(C) correlations, we note that the thermodynamic driving force for crystallization is the excess of the theophylline chemical potential in the solution over that in the crystal $\Delta \mu =\mu_{solution}-\mu_{crystal}$. Ignoring nonideality is reasonable for the electrically neutral theophylline molecules dissolved in n-octanol at moderate concentrations. We get $\mu_{solution}=\mu_{solution}^{0}+k_{B}T\;\ln\;C$ and ${\;\mu }_{crystal}=\mu_{solution}^{eq}=\mu_{solution}^{0}+k_{B}T\;\ln\;{\;C}_{e}$, so that $\Delta \mu /k_{B}T=\ln\left(C/C_{e}\right)$.

**Figure 3:**
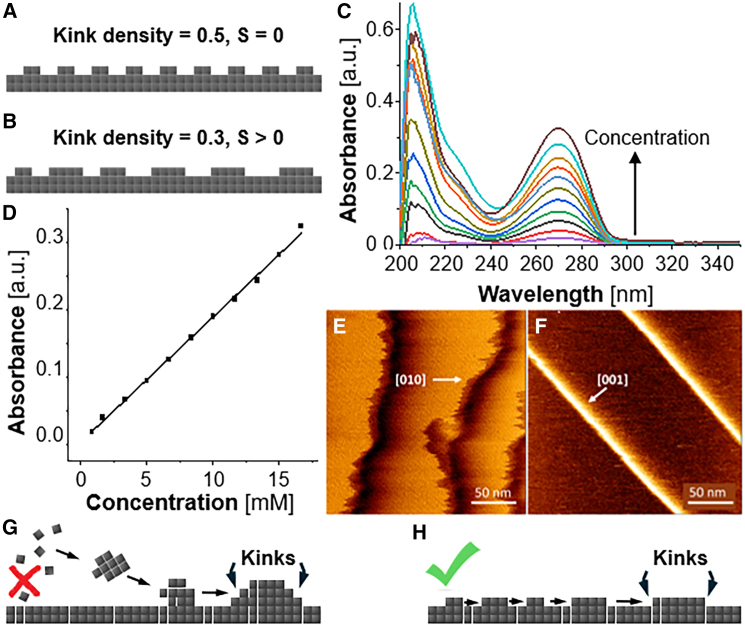
Potential mechanisms of nonlinear v(C) in the [001] direction (A and B) Schematic representation of step configurations with the maximum possible kink density of 0.5, which is unique and has entropy S = 0 in (A), and a structure with a kink density of 0.3, which has greater multiplicity and higher entropy in (B). (C) UV spectra of theophylline in n-octanol at concentrations ranging from 1 to 17 mM. (D) The absorbance at λ = 270 nm as a function of theophylline concentration. (E and F) High resolution images of the edges a [010] step, in (E), and a [001] step, in (F). (G and H) Schematics of two mechanisms of kink generation on a flat step edge. (G) By association of 2D solute clusters pre-formed on the terraces in front of steps. (H) By one-dimensional nucleation of new molecule rows.

Crystal growth constitutes a bimolecular chemical reaction between kinks and solute molecules. Correspondingly, the rate of this reaction depends linearly on the concentrations of kinks and solute.^24^ Kinks are located along steps, and the kink concentration is represented by kink density, i.e., the number of kinks per unit step length; if the step length is measured in the number of molecules, the kink density is dimensionless and equal to the reciprocal average number of molecules between kinks. The kink geometry dictates that the kink density is limited from above.^23^^,^^59^^,^^63^ Such a limit can be demonstrated by a configuration with a kink density of 0.5, in which two molecules of a new row along the step alternate with two vacancies (Figure 3A). This configuration, however, would have zero entropy.^59^^,^^63^ Careful evaluation indicates that maximizing the step entropy enforces more disordered configurations, for which the upper limit of kink density drops to ca. 0.3^59^^,^^63^^,^^64^^,^^65^^,^^66^^,^^67^ (Figure 3B). For many systems with relatively low kink energy kinks are readily generated and the upper thermodynamic threshold is reached when the crystal is in equilibrium with the solution.^9^^,^^59^^,^^63^^,^^68^ Thus, increasing supersaturation does not induce greater kink density and the step velocity is only driven by the chemical potential of the solute in a quasi-first order reaction.^24^ The rate of first-order reversible chemical processes scales with [exp(*Δμ/k*_*B*_*T*)−1],^69^ which, in view of the Δμ(C) relation above transforms to [exp(*Δμ/k*_*B*_*T*) − 1] = $\left(C/C_{e}-1\right)$. The linear v(C) correlation for steps growing in the [010] direction (Figure 2D) complies with the prediction of this model that assuming high kink density at equilibrium which stays constant at increasing supersaturation.

To understand the superlinear v(C) correlation for steps growing in the [001] direction, we first probe whether it may root in a preference for the incorporation of dimers, which, however, represent a minority solute population. Dimers in equilibrium with a majority of monomers have been shown to enforce quadratic v(C) for crystals of olanzapine and etioporphyrin I.^16^^,^^57^^,^^58^ Dimerization in olanzapine solutions was observed by Raman spectroscopy, whereas in solutions of etioporphyrin I, by UV-vis absorption spectroscopy. The strong Raman response of n-octanol prevents the Raman characterization of the theophylline solutions. The UV absorption spectrum of theophylline in n-octanol preserves its shape with increasing concentration (Figures 3C and 3D). Specifically, we observe no broadening or red shifts of the peaks. We conclude that no dimerization or higher order oligomerization of the solute occurs in the concentration range from 1 to 20 mM, in which the step velocities in [010] and [001] directions were measured (Figures 2D and 2E) and the solute remains in monomeric form. Thus, the analytical concentration of theophylline represents the concentration of monomers in the solution, and solute dimerization or oligomerization is not the cause of the observed superlinear increase of the step velocity in the [001] direction with solute concentration (Figure 2D).

Turning back to the kink density along steps growing in the [010] and [001] directions, high-resolution AFM imaging of crystal surfaces in equilibrium with the solution reveals that, as expected, [010] steps, whose v(C) correlation is linear (Figure 2D), appears very rough, indicating high kink density (Figure 3E). By contrast, steps growing in the [001] direction, which exhibit superlinear v(C) correlation, appear smooth, with very low kink density (Figure 3F). If the kink density at equilibrium with the solution is lower than the thermodynamic limit, increasing solute concentration drives higher kink density. As a result, the bimolecular reaction between kinks and solute, leading to incorporation into kinks, appears as a second order reaction with respect to the solute concentration and v(C) increases superlinearly (Figure 2E).

Two mechanisms by which more kinks may be generated in supersaturated solutions have been discussed (Figures 3G and 3H).^70^ The first mechanism involves 2D clusters of several molecules that form on the terraces between steps, diffuse along the terraces, and associate to the steps^68^ (Figure 3G). The association of 2D clusters to the steps produces several unfinished molecular rows, which terminate at multiple kinks.^68^^,^^70^ Since neither multiple kinks nor 2D clusters have been observed during the growth of theophylline steps, we find this mechanism unlikely; this mechanism may be exclusive to crystals for which the solute molecules reach the steps after adsorption on the terraces. A second mechanism relies the one-dimensional nucleation of new molecular rows (Figure 3H).^71^ Notably, one dimensional nuclei cannot be defined thermodynamically through the balance of the excess free energy of the two end molecules in a row and the free energy loss due to the formation of molecular contacts in the row.^72^^,^^73^ They can, however, be defined kinetically, as molecular rows of length such that their probability to grow is equal to their probability to dissolve (Figure 3H).^71^ Several analytical models based on these assumptions consistently predict that the step velocity v would scale superlinearly with C.^71^^,^^74^^,^^75^ The correspondence of this functional form to the measured v(C) for steps growing in the [001] direction suggests that the observed superlinearity may be due to enhanced kink generation at higher supersaturations.

### The anisotropy of the kink density

Distinct velocities of steps growing in different directions on a shared crystal face are commonly observed as noncircular dislocation spirals or elongated islands generated by two-dimensional nucleation.^14^^,^^15^^,^^51^^,^^76^ Typically, the step growth anisotropy is attributed to distinct kinetics of the incorporation of solute molecules into kinks of divergent orientation, whereas the kink density is assumed to be at the thermodynamic limit at all step orientations. In support of this explanation, the step shape anisotropy is typically independent of supersaturation^14^^,^^15^^,^^27^^,^^51^^,^^56^^,^^76^ and, in some cases, the kink density along steps of distinct orientations has been shown to equal the thermodynamic upper threshold.^15^

The distinct kink density along the two types of steps on the (200) face of theophylline crystals is somewhat unusual.^10^ Since the kink density ${\bar{n_{k}}}^{-1}$ ($\bar{n_{k}}$, average number of molecules between kinks) along steps in equilibrium with the solution is fully governed by the kink energy ω, ${\bar{n_{k}}}^{-1}=2{\left[\exp\left(\omega /k_{B}T\right)+2\right]}^{-1}$ (where $k_{B}$ is the Boltzmann constant, and T, temperature)^9^^,^^59^^,^^63^ this observation implies distinct energies of kinks along the [010] and [001] steps, with the kink energy on the [010] sufficiently low, ca. 4 kJ mol^−1^, to ensure the observed high kink density. The kink energy is the net sum of the energies of the molecular bonds that break and reconstitute when a kink is formed.^9^^,^^68^^,^^77^ In turn, the half sum of all bonds in the crystal lattice constitutes the crystallization enthalpy.

**Figure 4:**
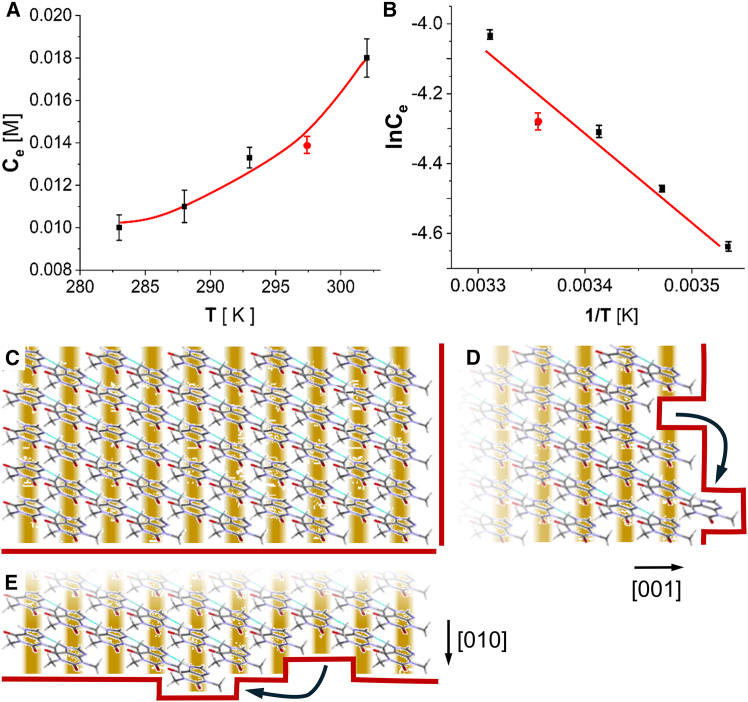
Crystallization thermodynamics and the kink energy (A) The dependence of the solubility $C_{e}$ of anhydrous theophylline crystals in n-octanol on temperature. Squares, the spectroscopically determined terminal concentrations of theophylline crystals growing in n-octanol. Circle, the concentration at which steps monitored by AFM do not grow or dissolve but instead fluctuate around fixed locations. (B) The $C_{e}\left(T\right)$ data from (A) in van ‘t Hoff coordinates. Error bars in (A) and (B) represent standard deviations from the averages of five independent measurements. (C) Schematic representation of the structures of kink-free steps facing [010] and [001] directions. C atoms are shown in gray; N, in magenta; O, in red; H, in silver. Blue lines depict H-bonds between imidazole N7 and N9 of adjacent molecules. Brown stripes highlight stacks of molecules bound by π−π stacking interactions. Red contours highlight step edges. (D and E) Schematics of the creation of kinks by removing a molecule from the step edge and positioning it next to a [001] step in (D) and a [010] step in (E). Red contours highlight step edges and newly created four kinks.

The relation between kink energy and crystallization enthalpy $\Delta H_{cryst}^{o}$, also called latent heat of crystallization, suggests a test of the feasibility of the hypothesis that the anisotropy of the kink density on theophylline steps is due to divergent kink energy. We evaluate $\Delta H_{cryst}^{o}$ from the temperature dependence of the solubility $C_{e}$ (Figures 4A, 4B, S7, and S8) using standard thermodynamic relations $K_{cryst}=C_{e}^{-1}$, $\Delta G_{cryst}^{o}=-RT\;\ln\;K_{cryst}$, and van ‘t Hoff’s law $\partial \ln\;C_{e}/\partial \left(T^{-1}\right)=\Delta H_{cryst}^{\;o}/R$, and assuming, again, that the activity coefficients of the electrically neural theophylline molecules are close to unity.^27^^,^^32^^,^^78^ The C(T) data (Figures 4A and 4B) reveal that $\Delta H_{cryst}^{\;o}$ = −20.5 ± 0.6 kJ mol^−1^, $\Delta S_{cryst}^{\;o}$ = −34.0 ± 0.4 J mol^−1^K^−1^, and $\Delta G_{cryst}^{\;o}$ = −10.5 ± 0.7 kJ mol^−1^ at 298 K. The measured value of $\Delta H_{cryst}^{\;o}$ roughly sums the energies of all kinks on all faces of a crystal. It is consistent with assuming that the energy of kinks along [010] steps on (200) faces is sufficiently low to yield closely spaced kinks, whereas the energy of the kink along the [001] step is higher and only allows low kink density.

The molecular structure of a (200) plane (Figure 4A) illuminates why the kinks on [010] and [001] steps have divergent energies. In the crystal lattice, the molecules are bound by hydrogen bonds between the nitrogens in the imidazole ring that form chains along the [011] direction.^79^ A second chain of strong π-π stacking interactions extends in the [010] direction (Figure 4C). Weaker van der Waals bonds form with other functional groups of the adjacent molecules in and out of the depicted plane.^79^ To evaluate the kink energy, we follow a thermodynamic pathway put forth by Burton et al*.*^9^ Moving a molecule embedded in a [001] step edge to a position along the same step breaks two π−π stacking bonds, one H-bond, and several van der Waals contacts (Figure 4D). Depositing the molecule at an intact segment of the step edge restores an H-bond; the bonds with molecules in the underlying (200) plane are identical in the initial and starting configuration. In balance, the cost of the created four kinks is the energy of two π−π stacking bonds, which can be high and enforce the low kink density on [001] steps, for which at supersaturation an increase in the step roughness was observed (Figure S9). By contrast, the same operation on a [010] step (Figure 4E) breaks one π−π stacking and one H-bond, but when the extracted molecule is positioned at the step edge, it binds with equivalent one π−π stacking and one H-bond. Thus, the cost of the four created kinks on a [010] step is limited to the energy of van der Waals contacts in the (200) plane. The low energy of these kinks underpins their high density.

### Control of crystal morphology by varying supersaturation

Theophylline crystals exhibit a birefringence of 15%, higher than that of calcite or quartz.^6^ These crystals find application as polarization-sensitive optical elements for holography. The crystal shape that provides maximum in-plane optical anisotropy is sheets parallel to the (200) crystal plane. Such crystals grow readily from several organic solvents. Since the crystal habit is dominated by the slowest growing faces,^44^^,^^62^ this shape implies that the (200) face growth rate R is slower than the growth rate of the adjacent (010) and (001) faces. We attribute the slow growth rate of (200) faces to the chains of hydrogen and π-π stacking bonds that populate the (200) plane (Figures 4C–4E).^79^ The (200) faces grow by the spreading of layers (Figure 1B), and the strong broken bonds at the edges of unfinished (200) layers enforce a high surface free energy γ of the layer edges. In turn, the high surface free energy drives up the free energy barrier for the 2D nucleation of new layers $\Delta G_{2D}^{*}=\pi \Omega h\gamma^{2}\Delta \mu^{-1}$, where Ω is the molecular volume and h is the layer thickness,^27^^,^^72^ and slows down layer generation. The face growth rate $R=hvl^{-1}$, where l is the separation between steps and $l^{-1}$ is the step density.^9^ Thus, suppressed layer generation due to high γ sensitively decreases $l^{-1}$ and slows down face growth.

**Figure 5:**
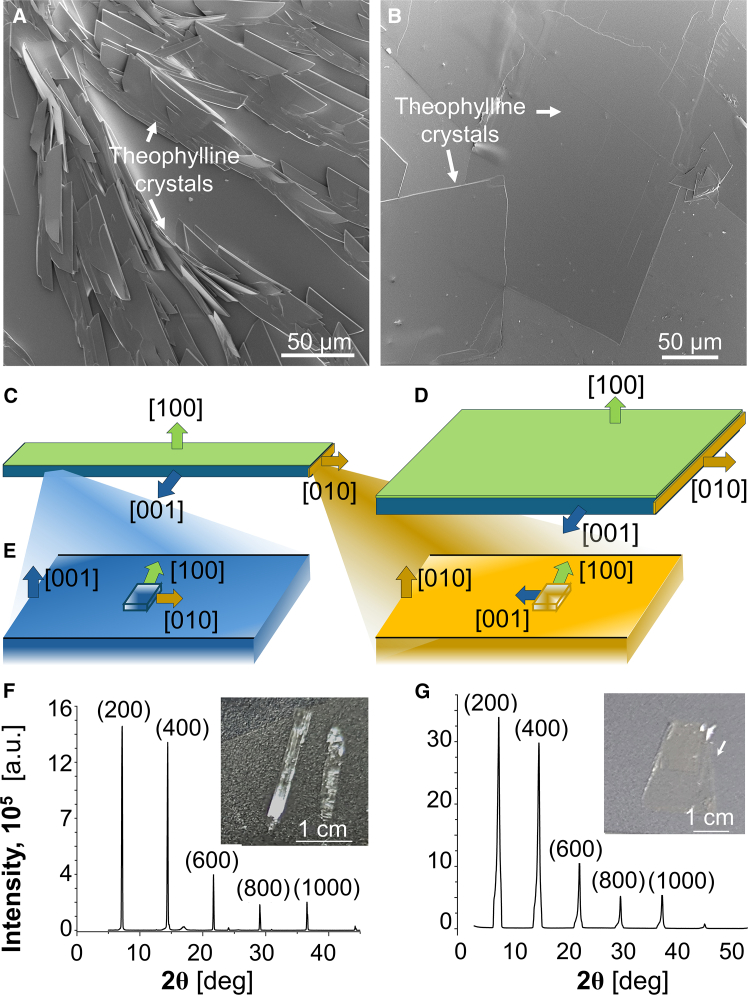
Control of crystal morphology (A and B) Scanning electron microscopy images of theophylline crystals grown from a 14 mM octanol solution in (A), and from a 20 mM solution in (B). (C and D) Schematic representations of the relation between face size and growth rate in normal direction. (E) Schematic of 2days islands on (001) and (010) faces and the orientations of their edges. (F and G) Morphology control during growth from chloroform solutions. X-ray diffractograms show single orientation of {100} planes parallel to the (200) surface for crystals grown from 27 mM solution in (E) and 83 mM in (F). Insets: Representative photographs of crystals grown under respective conditions.

We use the insights on the crystal growth mechanisms obtained above to control the morphology of the theophylline sheets. We show that we can direct the growing crystals to either ribbon-like or slate-like shapes. At low supersaturation (C = 14.5 mM) the theophylline sheets present as ribbons elongated in the [010] direction (Figure 5A), indicating that the growth of the (001) faces is suppressed (Figure 5C). By contrast, sheets grown at higher supersaturations, at C = 20 mM and above, are isometric in the [010] and [001] directions (Figure 5B), manifesting comparable growth rates of the (010) and (001) faces (Figure 5D). The supersaturation dependence of the crystal morphology runs parallel to the supersaturation response of the step growth anisotropy on the (200) face (Figures 2D and 2E). The analogous responses to the supersaturation of R and v anisotropies do not constitute a trivial observation since the growth rates R of each face is determined by fully independent pairs of step velocities v and densities $l^{-1}$. Similar parallel shifts of the layer and crystal anisotropies were observed with calcite crystals, selectively inhibited by chiral amino acids.^80^

The strong supersaturation dependence of the growth rate of the (001) face suggests that on that face, the barrier for the 2D nucleation of new layers $\Delta G_{2D}^{*}$ is dominated by high γ. At low supersaturations Δμ the resulting high $\Delta G_{2D}^{*}$ suppresses layer generation and face growth. The high value of γ forces high sensitivity of $\Delta G_{2D}^{*}$ to increasing Δμ, which lowers the barrier and allows fast growth at higher supersaturations. We relate the high surface free energy of the edges of the layers on a (001) face to the crystal structure and the strength of the crystal bonds (Figures 4C–4E). The edges of a new layer nucleus on a (001) face would be decorated with dangling π-π stacking bonds in the ⟨010⟩ directions (Figures 5E and 4D) and weaker van der Waals contacts in the ⟨100⟩ directions; the π−π stacking bonds would contribute to high γ. By contrast, a new layer nucleus on a (010) face is surrounded by broken weak van der Waals contacts in both ⟨001⟩ and ⟨100⟩ directions (Figures 5E and 4E). In consequence, its surface free energy γ would be lower, inducing a lower nucleation barrier $\Delta G_{2D}^{*}$ at lower supersaturation, which, however, is less sensitive to supersaturation. The resulting R of the (010) face would be relatively high at low supersaturation and only moderately increase with higher Δμ.

To expand the scope of the current study, we show that the principle of using the crystallization mechanisms to design morphology control strategies applies to other organic hydrophobic solvents such as chloroform. As in n-octanol, crystallization from solution with different supersaturations yields crystals with distinct morphologies. During growth from chloroform, crystallization from a 27 mM solution yields needle-like crystals as the crystals grow rapidly along the **b**-axis (Figure 5F). The higher concentration of 83 mM results in 2D crystalline sheets (Figure 5G). Again, theophylline packs into the same orthorhombic polymorph (Pna2_1_) as shown by single crystal X-ray diffraction and pXRD (Figures 5F, 5G, and S10–S13).

### Design of crystal self-regeneration strategies

**Figure 6:**
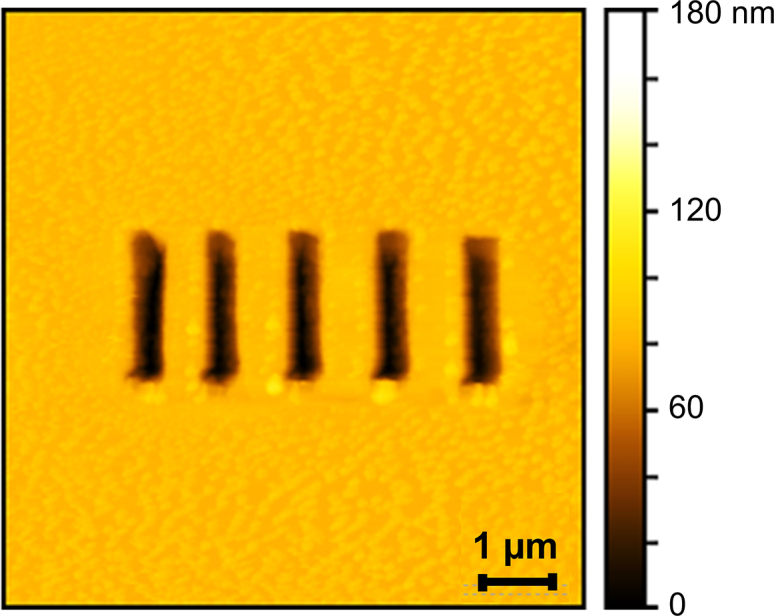
Line grating created by scanning thermal lithography

Certain optical applications require etching grooves and gratings on the surfaces of theophylline crystals.^6^ Such patterns can be etched by scanning thermal lithography, localized sublimation by the AFM tip, a technique that avoids charging damage and carbonization defects (Figure 6). The insights into the growth mechanism of theophylline crystals, namely, that they grow classically, by the incorporation of single solute molecules, and on the kink density anisotropy, allow us to fully restore the surface of a crystal, even if grown six months ahead, after etching. In this way, the surface may be recycled and reused by the local application of a supersaturated solution of theophylline, avoiding the lengthy process of crystal dissolution and regrowth.

**Figure 7:**
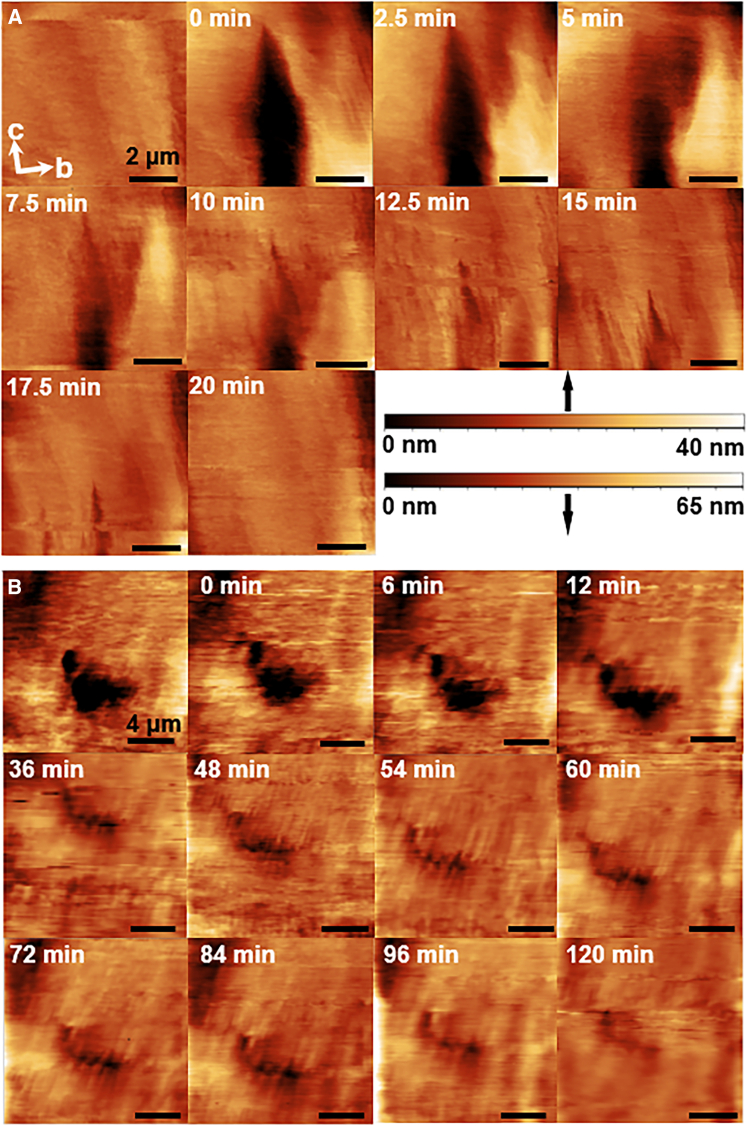
Regeneration of grooves etched in the (200) crystal surface after exposure to 18 mM theophylline solution monitored *in situ* by time-resolved AFM. Times after the start of regeneration indicated on images (A) Groove elongated along [001] or c direction. (B) Groove elongated along [010] or b direction.

The anisotropy of the kink density along the edges of unfinished layers on the (200) theophylline crystal face guided the design of a strategy to select the orientation of grooves etched on the crystal surface. We etched a groove by heating the AFM cantilever, which led to the local sublimation of the theophylline molecules. We then introduced a supersaturated solution of theophylline in n-octanol. Grooves elongated along the **c** direction, which heal owing to growth in the [010] direction, recover perfectly (Figure 7A). We attribute the perfect alignment of the newly grown and the existing crystal matter to the high kink density along [010] steps (Figure 3E), which allows steady and uniform growth of the two walls of the groove facing each other. By contrast, grooves etched in the **b** direction, which heal by growth of kink-poor [001] steps, fill up imperfectly, leaving voids and crevices (Figure 7B). The 3D images of the process are shown in Figures S14 and S15. The paucity of kinks, combined with the competition for dampened solute between the opposing groove walls induces instabilities that lead to the growth of protrusions separated by voids. The local surface roughness changes of the crystals were measured to quantify and compare the regeneration extent of different crystal faces, see Figure S17.

## Discussion

We demonstrate that an in-depth understanding of crystal growth mechanisms can support the development of novel and more efficient methods to tailor materials properties for improved performance. We show that driving crystallization toward classical growth modes, by using a solvent dominated by weak intermolecular forces, grants the ability to control crystal morphology. We design a strategy to transition between needle-like and 2D slate-like crystal shapes by the simple adjustment of the supersaturation. We attribute the distinct supersaturation dependences of the growth rates of adjacent faces to the strengths of the bonds severed at the edges of the 2D islands on each face that govern the surface free energies of island edges and the barriers for the nucleation of new crystal layers. Moreover, we show that crystals that grow through classical growth mode can heal and regrow after cleavage and damage. Thus, driving the crystallization toward a classical growth mode enables the manipulation of growing crystals toward desired morphologies, self-regeneration of molecular crystals with low molecular mobility, and, potentially, polymorph specificity.

### Limitations of the study

The mechanisms of kink generation should be validated by fast-scan *in situ* AFM observations at near-molecular resolutions that allow the visualization of individual kinks and their dynamics in real time.

Molecular dynamics and density functional theory computations should be applied to quantify the strengths of the lattice bonds, whose dynamics govern the generation of kinks, and the consequences of the bond strength and dynamics for the crystal shapes and evolutions of the crystal features.

The demonstrated strategy to control the crystal’s shape and dimension should be tested with other crystalline materials and pharmaceuticals.

## Resource availability

### Lead contact

Requests for further information and resources should be directed to and will be fulfilled by the lead contact, Peter G. Vekilov (vekilov@uh.edu).

### Materials availability

This study did not generate new unique reagents.

### Data and code availability

• All data reported in this article will be shared by the lead contact upon request.
• This article does not report original code.
• Any additional information required to reanalyze the data reported in this article is available from the lead contact upon request.

## Acknowledgments

This work was supported by the National Science Foundation (Award No. DMR-2128121), the Welch Foundation (Award E-2170 and the Welch Center for Advanced Bioactive Materials Crystallization, Award V-E-0001), and the Minerva Foundation. AE acknowledges support from the Weizmann Institute "Advancing Women in Science" postdoctoral fellowship program.

## Author contributions

A.N.-E^.^ conceived this study, carried out most experiments, and wrote the first draft of the text; H.-J.L. carried out high-resolution AFM imaging; S. M. and M. Y. took part in AFM data collection; I.R.-G. took part in thermal scanning probe lithography (TSPL) data collection and analysis; M.P. and G. F. designed the TSPL instrument and provided technical support with it; A. K. took part in powder XRD measurement and analysis; Y.F. took part in powder XRD measurement and analysis; Y.D.-P. solved the single crystal structure and did phase indexing analysis; D. O. provided crucial funding and supervised experiments; P.G.V. supervised the experiments, provided data interpretation, and wrote the text.

## Declaration of interests

The authors declare no competing interests.

## STAR★Methods

### Key resources table

**Table:** 

REAGENT or RESOURCE	SOURCE	IDENTIFIER
**Chemicals, peptides, and recombinant proteins**		

Theophylline	Sigma aldrich	CAS: 58-55-9
Chloroform	Sigma aldrich	CAS: 67-66-3
n-Octanol	Sigma aldrich	CAS: 111-87-5

**Software and algorithms**		

Gwyddion Version 2.62	Czech Metrology Institute	http://gwyddion.net/; RRID:SCR_015583
NanoScope Analysis v1.40r1	Bruker	http://nanoscaleworld.bruker-axs.com/nanoscaleworld/media/p/775.aspx; RRID: SCR_026153
CCDC Mercury		https://www.ccdc.cam.ac.uk/solutions/software/mercury/
SHELXT	G. M. Sheldrick	https://doi.org/10.1107/S2053273314026370; RRID: SCR_014220
SHELXL Version 2014/7.	G. M. Sheldrick	https://doi.org/10.1107/S2053229614024218; RRID: SCR_014220
APEX	Bruker	https://www.bruker.com/en/products-and-solutions/diffractometers-and-x-ray-microscopes/single-crystal-x-ray-diffractometers/sc-xrd-software/apex.html?utm_source=Advertising&utm_medium=GoogleAd&utm_campaign=BBIO-Industrial-AIC-NMR-IndustrialPMax-H1-2025&gad_source=1&gad_campaignid=22400402808&gbraid=0AAAAADsq6H0egjoymFrihMhdyyqm9CX_-&gclid=Cj0KCQjww-HABhCGARIsALLO6XzOKGWL8fo48ziMYB3xP7LdirTvygsh8k86SLl7v8-3dGzOLAYBqeEaAkGREALw_wcB
MDI JADE 6.0 Pro	Materials Data	http://www.icdd.com/mdi-jade

### Method details

#### Reagents and solvents

Solvents and reagents were purchased from commercial sources and used as received, unless otherwise indicated. For all aqueous mixtures, double-distilled water (TDW) was used (Barnstead NANOpure Diamond water system). Organic solvents for spectroscopic and microscopic studies were of HPLC grade. 1,3-dimethylxanthine (theophylline)) was purchased from Sigma-Aldrich and used without further purification.

#### Solutions

Theophylline solutions in n-octanol were prepared by dissolving theophylline powder (48 – 75 mg) in 20 ml of n-octanol, the mixture was heated to 100°C and mixed by magnetic stir bar. The hot solution was filtered using PTFE syringe filter with 0.2μm pour size. Supersaturated theophylline solutions in CHCl_3_ were made by dissolving theophylline powder (10 – 40 mg) in 20 ml of n-octanol, the mixture was heated to 60°C and mixed by magnetic stir bar. The hot solution was filtered using PTFE syringe filter with 0.2 μm pour size.

#### Crystal growth

The supersaturated solutions were placed in a crystallization incubator at 22°C (Molecular Dimensions, INC. Model MD5-601) to allow crystallization. Theophylline crystals were grown either from pure n-octanol or from CHCl_3_. The mature crystals were deposited on a glass slide with a small amount of solvent to let the crystals adhere to the glass surface.

#### Solubility determination

The solubility was measured by steady state absorbance spectroscopy. Different amounts (70 mg, 60 mg, and 50 mg) of theophylline were dissolved octanol (20 ml), the vials were incubated in a crystallization incubator each batch at a different steady temperature (10°C, 15°C, 20°C, 27°C) for at least one month for precipitation. The solubility was determined when the absorbance for different amounts of initial theophylline had the same steady absorbance spectrum, as the solubility in equilibrium is independent of the amount of precipitate and the initial supersaturation concentration (Figure S8).

#### Powder X-ray diffraction

The pXRD measurements were carried out in reflection mode using a TTRAX III (Rigaku, Japan) diffractometer equipped with a scintillation detector and a rotating Cu anode operating at 50 kV and 200 mA in Bragg–Brentano geometry. In-plane XRD measurements were performed on a SmartLab (Rigaku, Japan) diffractometer, equipped with a rotating Cu anode operating at 45 kV and 200 mA and a HyPix-3000 2D detector in in-plane geometry with an incident slit of 0.1 mm. During the measurement the source and the detector were at theta-2theta 0.3°. The parallel beam was shaped by a 0.5° in-plane parallel slit analyzer (PSA) before and after the sample. The detector operated in 0D mode with scattering and receiving slits open. Diffraction patterns were measured at a step size of 0.016°.

To probe side faces of the crystal in-plane 2-theta angle was set to the value specific for the plane of interest and azimuthal phi-scan was performed.

#### Scanning electron microscopy (SEM) imaging

We used a Zeiss Supra 55 FEG-SEM or Zeiss Ultra 55 FEG-SEM operating at 1-20 kV. Images were obtained using working distance (WD) of 3-5 mm, and a standard aperture (30 microns).

#### AFM imaging

MultiMode atomic force microscope (Nanoscope VIII or IV; Bruker) in tapping mode was used to monitor the growth of crystals. To collect images, 500 μl of the prepared sample was injected into the AFM liquid cell over theophylline crystals that were grown on a glass substrate (0.5 mm) attached to a 15 mm metal disk (Ted Pella, Inc). To avoid any leakage, an O-ring was inserted firmly to the liquid cell. The temperature in the liquid cell reached equilibrium of 27.0 ± 0.1°C within 15 min, higher than room temperature (ca. 22°C), because of heating by the AFM scanner and laser. Height, amplitude, and phase images were collected in image sizes ranged from 0.5 μm × 0.5 μm to 50 μm × 50 μm, with scan rates ranging from 1 to 2 s^−1^ in most images.

Scans were made with a silicon tip on a silicon nitride cantilever SNL10 type C (Frequency 56kHz; spring constant k=0.24 N/m).

NanoRacer high-speed AFM from Bruker was employed to capture high-resolution images for the analysis of kink density along different crystallographic directions. These small scan area images were crucial for evaluating the distribution of kinks on the surface. The sample, consisting of a glass substrate with theophylline crystals, was affixed to the bottom of a sample holder using glue, and 1 mL of growth solution was subsequently added. Whole AFM system was placed in chamber maintained at room temperature throughout the imaging process. To minimize physical interference with the sample surface, tapping mode was applied. The scan equips with a USC-F1.2-k0.15-10 tip featuring a resonance frequency of 1.2 MHz and a force constant of 0.15 N/m. The scan areas ranged from 50 nm × 50 nm to 2 μm × 2 μm, with scan rates varying between 10 and 30 Hz.

#### Thermal heating of AFM Probe

To achieve local heating of the AFM tip, we employed a fast-scan AFM head that fit onto the base of a commercial MultiMode AFM system. This head offered the option for photothermal excitation. By increasing the voltage applied on the photothermal laser diode we could successfully generate local sublimation of the Theophylline crystal. Defects were generated in contact mode with the photothermal laser aligned on the cantilever, at scan rates of 0.5 Hz. The fast-scanning AFM head and control electronics were designed and built by Georg E. Fantner’s group, Ecole Polytechnique Fédérale de Lausanne, Lausanne, Switzerland.^81^ Images were acquired by the custom LabView based software from Georg E. Fantner’s group. Scans and defects were made with a silicon tip on a silicon nitride cantilever (Bruker FASTSCAN-B). All images were analyzed using Gwyddion 2.62 software.^82^

#### Kinetic AFM measurements

The dependence of the step velocity on the step height was measured by monitoring the advancement of steps with different heights in the same crystalline layered stack towards a reference point on the surface. We found that all steps advance with a steady velocity and that the step velocity is independent of the step height, thus indicating a mechanism of direct incorporation of monomers to the grown crystal rather than growth via monomer adsorption and subsequent surface diffusion to the kink site.

#### X-Ray crystal structure analysis

Crystal data: C7H8N4O2, colorless plate, 0.184 x 0.052 x 0.015 mm, Orthorhombic Pna21, a=24.2655(8)Å, b=3.76210(10)Å, c=8.4743(2)Å, a=b=g=90°, from 26938 reflections for 75° data, T=100(1)K, V=773.61(4)Å3, Z=4, Fw=180.17, Dc=1.547 Mg.m-3, m=1.000 mm^-1^.

Data collection and processing: Rigaku Synergy-R diffractometer equipped with HyPix-Arc 150 detector, CuKa (l=1.54184Å), -30≤h≤30, -4≤k≤4, -10≤l≤10, frame scan width = 0.25°, scan speed 1.0° per 0.64 sec, 26938 reflections collected, 1574 independent reflections (R-int = 0.0426). The data were processed with CrysAlisPRO.

Solution and refinement: Structure solved with SHELXT. Full matrix least-squares refinement based on F2 with SHELXL on 120 parameters with 1 restraint gave final R1 = 0.0390 (based on F2) and wR2 = 0.1056 for data with I>2s(I) and, R1 = 0.0419 and wR2 = 0.1090 on 1574 reflections, goodness-of-fit on F2 = 1.078 largest electron density peak 0.196 e.Å-3. Largest hole –0.217 e.Å-3.
