# Supplementary material for: Lattice bond chains administer kink anisotropy and inform strategies of theophylline crystal self-healing
Source: iScience. 2025 Jun 10;28(7):112866. doi: 10.1016/j.isci.2025.112866 (PMC12268690; doi:10.1016/j.isci.2025.112866)
Supplement: Document S1. Figures S1–S17 [file mmc1.pdf]

iScience, Volume *28*

Supplemental information

Lattice bond chains administer kink anisotropy and inform strategies of theophylline crystal self-healing

Angelica Niazov-Elkan, Huan-Jui Lee, Sima Mafi, Manasa Yerragunta, Irit Rosenhek-Goldian, Marcos Penedo, Georg Fantner, Anna Kossoy, Yishay Feldman, Yael Diskin-Posner, Dan Oron, and Peter G. Vekilov

Supplemental Figures

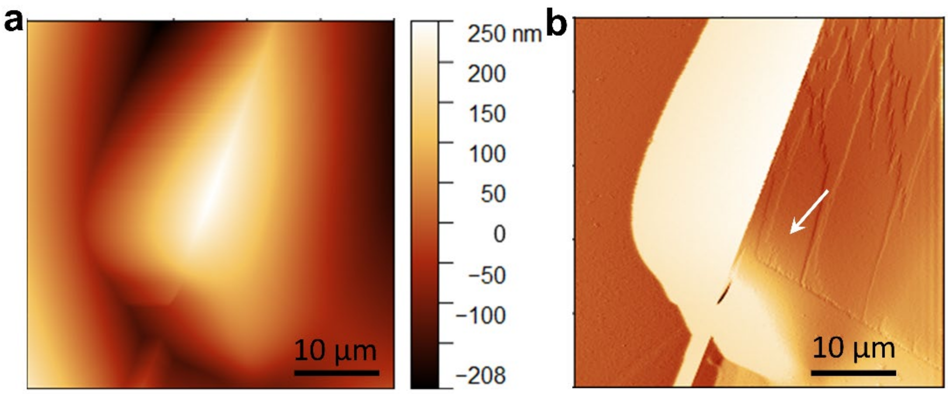

Figure S1. The origin of growth steps. (a) height, and (b) amplitude error, showing the possible steps origin at the crystal edge (marked by a white arrow).

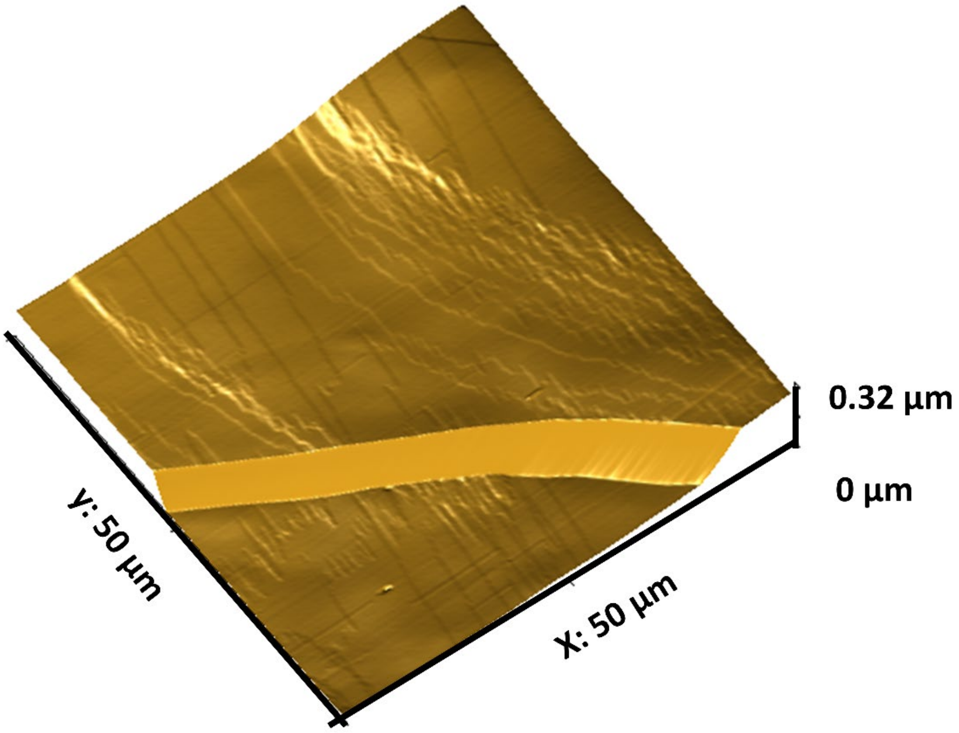

Figure S2. Single steps and macrosteps. AFM image with an overlay of topography and amplitude error, showing the growth of theophylline crystals from 0.015M solution.

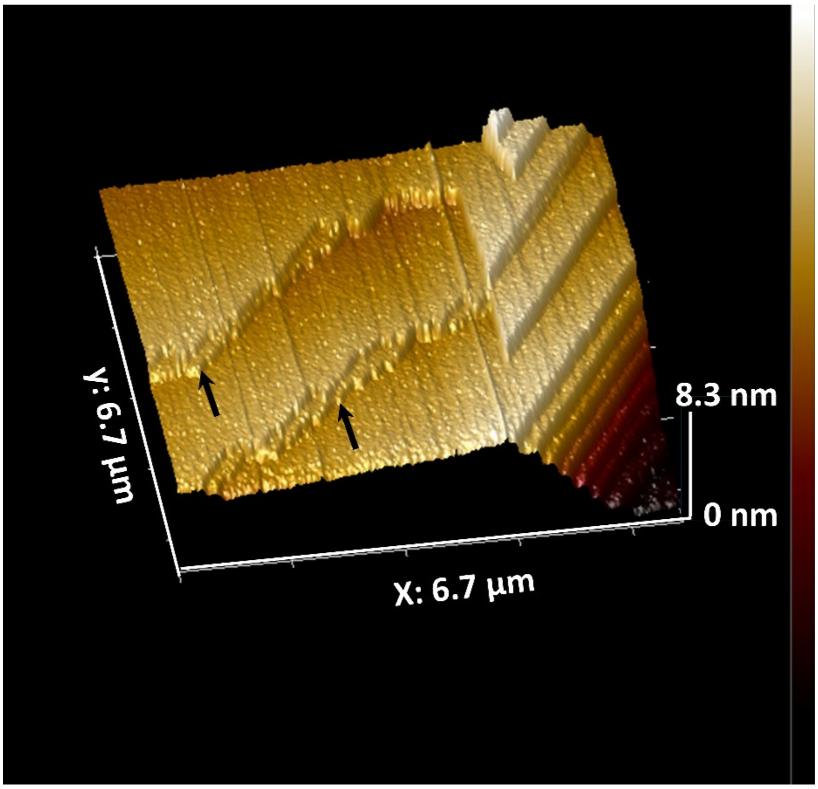

Figure S3. Simngle and double steps. 3D image of the growing theophylline showing two sub-steps of theophylline, each sub-step is~1.2nm, and the two form step with the height of one unit cell in the [100] direction.

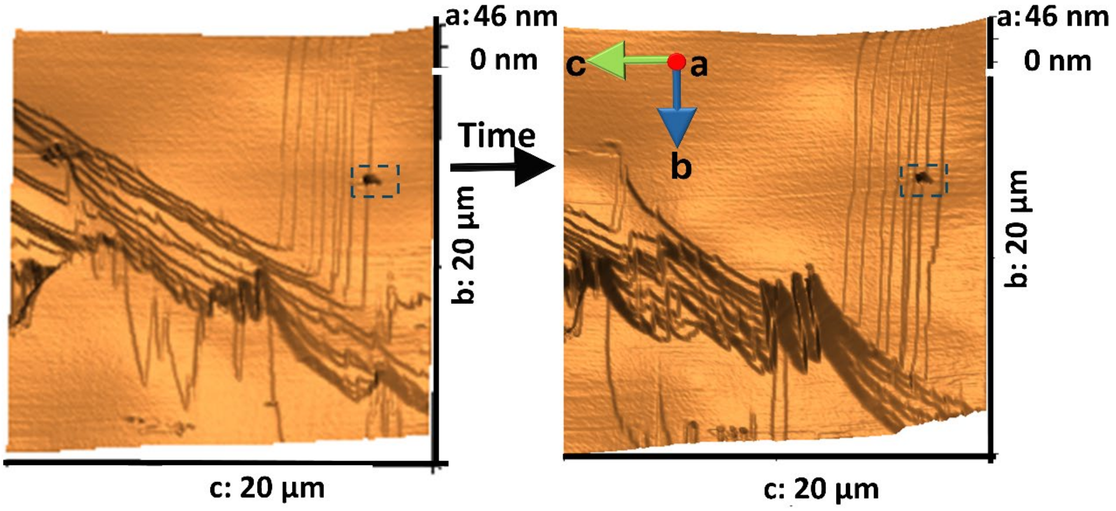

Figure S4. The evolutions of step locations. Representative, image of step velocity in the b and c direction measurement procedure relatively to a constant defect/ feature (marked with a dashed rectangle).

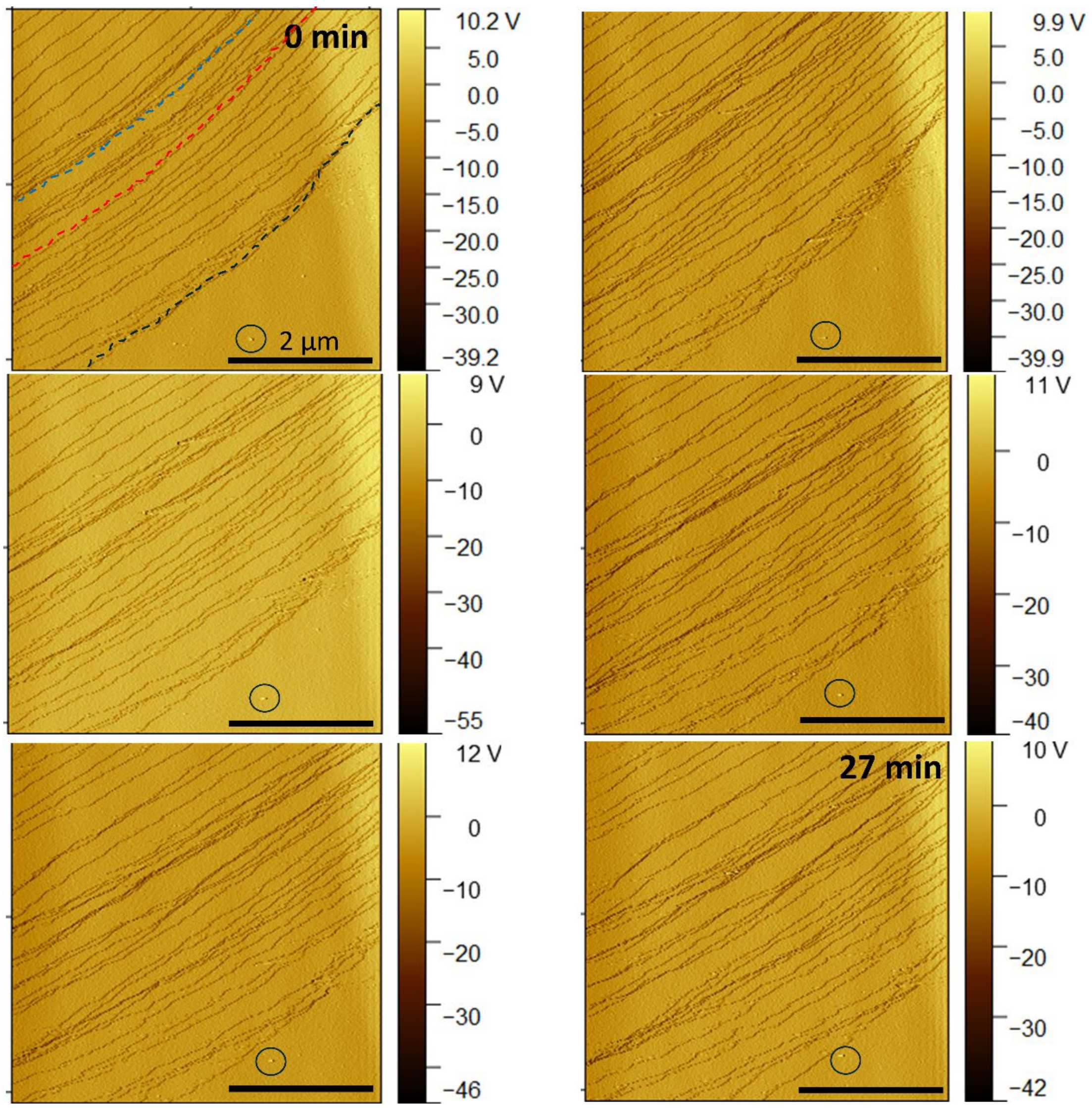

Figure S5. The growth of steps of different heights. AFM amplitude error images that show the advancement of steps at different heights (marked by red, blue, and black dashed lines), towards a non-mobile reference point (marked by a black circle)

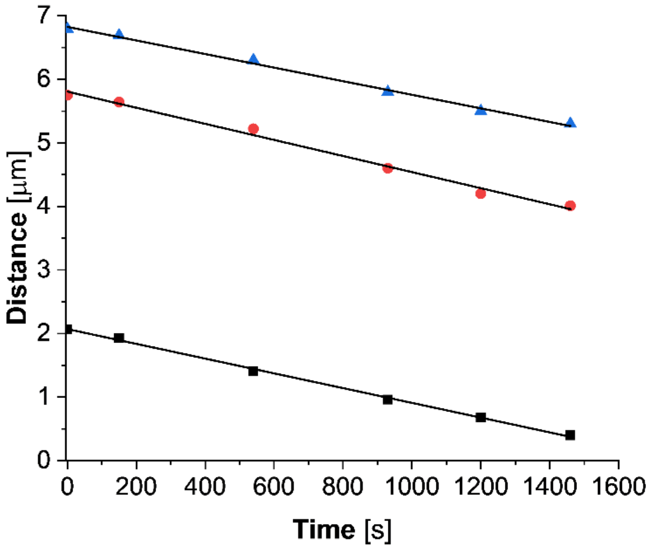

Figure S6. The advance of the steps towards a reference point. The three monitored steps grow at the same velocity. The blue, red, and black dots represent the velocity of the marked steps in Figure S3.

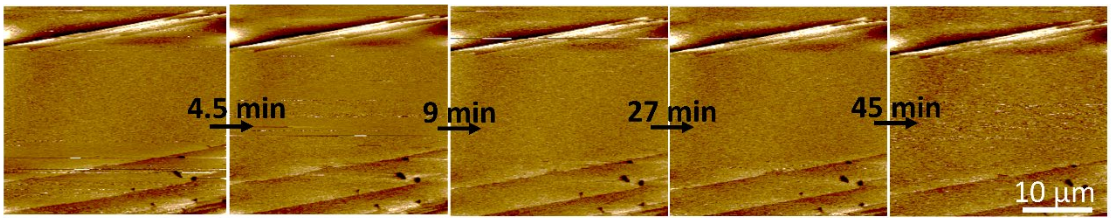

Figure S7. Steps at equilibrium between solution and crystal. AFM amplitude error image, showing theophylline crystal in equilibrium over time. At equilibrium concentration, the crystals change locally, they grow and dissolve in a way that there is no overall change, and overall, the crystals remain in a dynamic equilibrium.

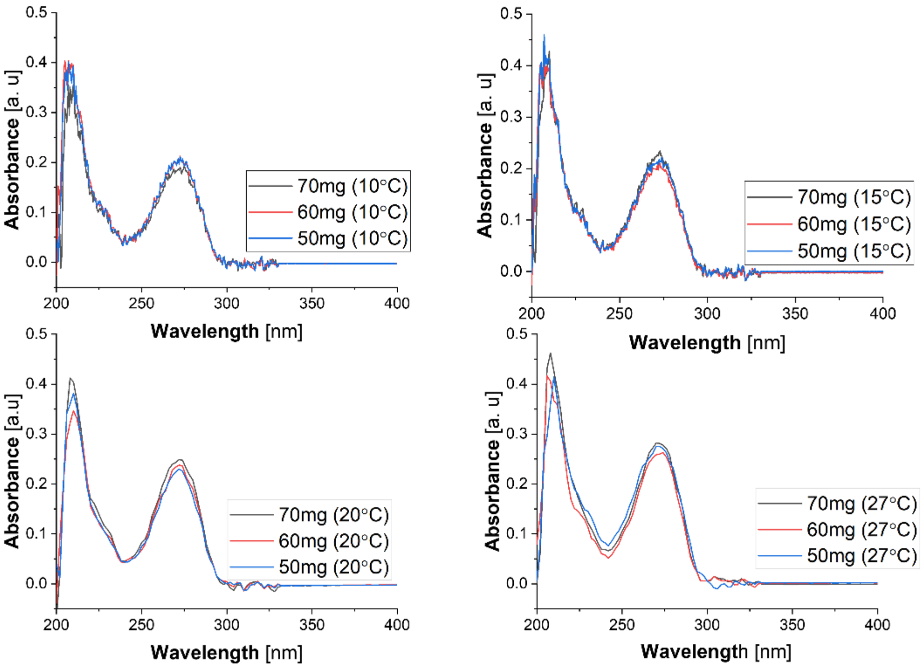

Figure S8. Determination of the solution concentration at equilibrium with crystals. The final concentration at each temperature is independent of the initial concentration, listed in the plots. (a) equilibrium absorbance at 10°C. (b) equilibrium absorbance at 15°C. (c) equilibrium absorbance at 20°C. (d) equilibrium absorbance at 27°C.

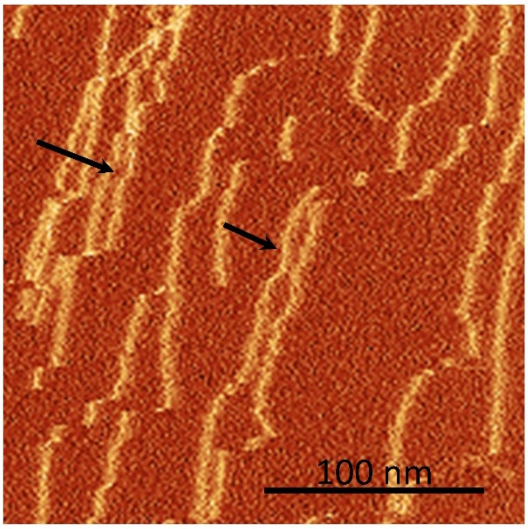

Figure S9. Determination of the step roughness. Amplitude error image of step roughness in supersaturation, at 0.167m. Growth is in the *c* (slow) direction

Needle-like crystals

$Pna2_1$

a=24.37A n_a= 1.47

b=3.76A n_b = 1.46

c= 8.44A n_c=1.7

α=β=Y=90°

V=774.67Å^3

2D crystalline sheets

$Pna2_1$

a=24.26A n_a= 1.47

b=3.76A n_b = 1.46

c= 8.47A n_c=1.7

α=β=Y=90°

V=773.612Å^3

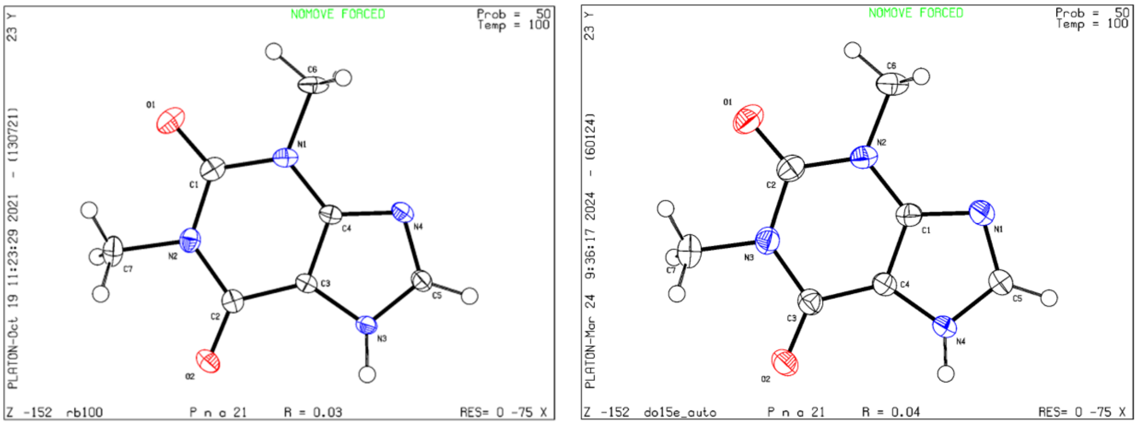

Figure S10. The structures of two theophylline crystals with distinct morphologies. ORTEP representation, showing that both needle-like (1D) and the crystalline 2D sheets that were grown from low (10%) and high (>30%) supersaturation concentrations have a single crystal form with the same crystal structure.

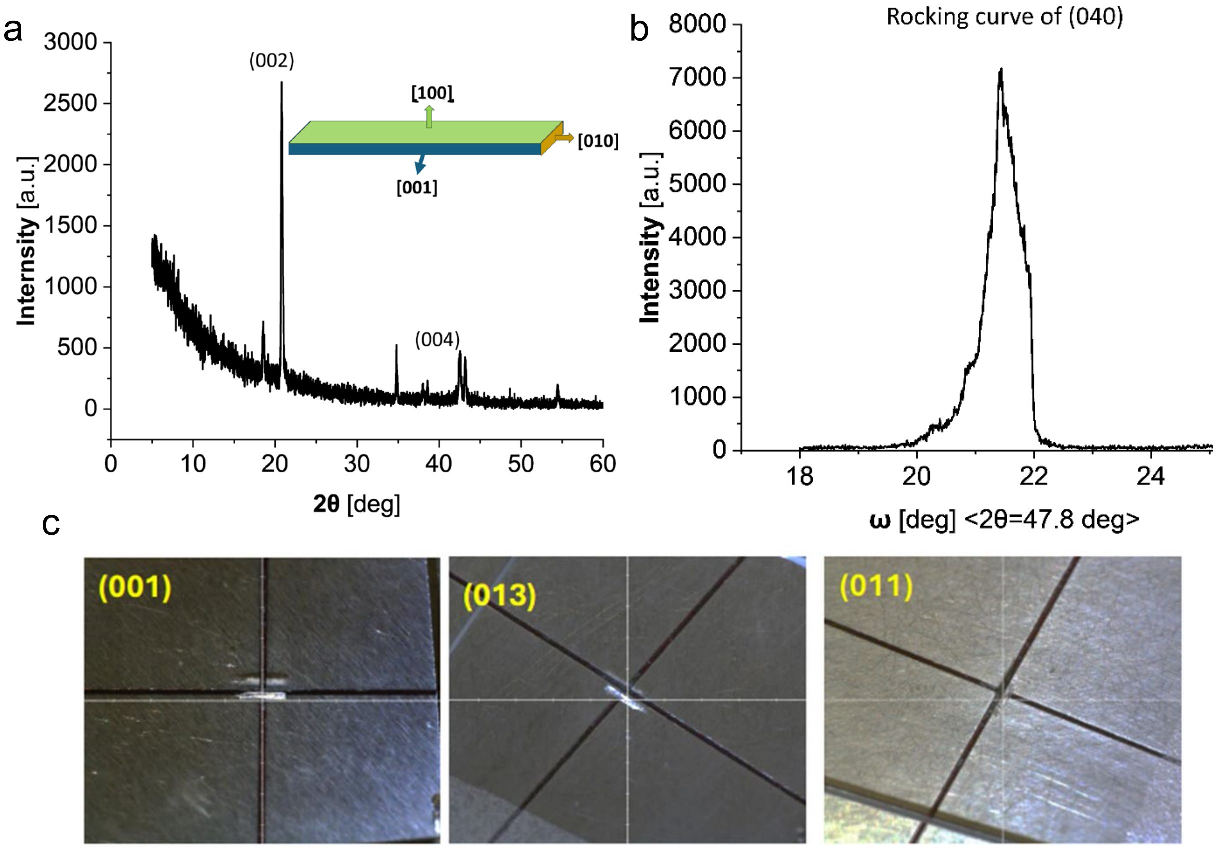

Figure S11. X-ray diffraction characterization of theophylline single crystals. Panels (a)-(c) elaborate on the orientation of faces for the crystal shown in Figure 9a: (a) 2ϑ/ ϑ in-plane diffractogram of longer crystal face shows {001} peaks (b) Rocking curve, ω scan, of (040) peak taken at shorter crystal face. (c) shows crystal orientation from Figure… with a certain plane parallel to dashed horizontal line as deduced from azimuthal scan while in-plane 2ϑ angle is set to that of the plane.

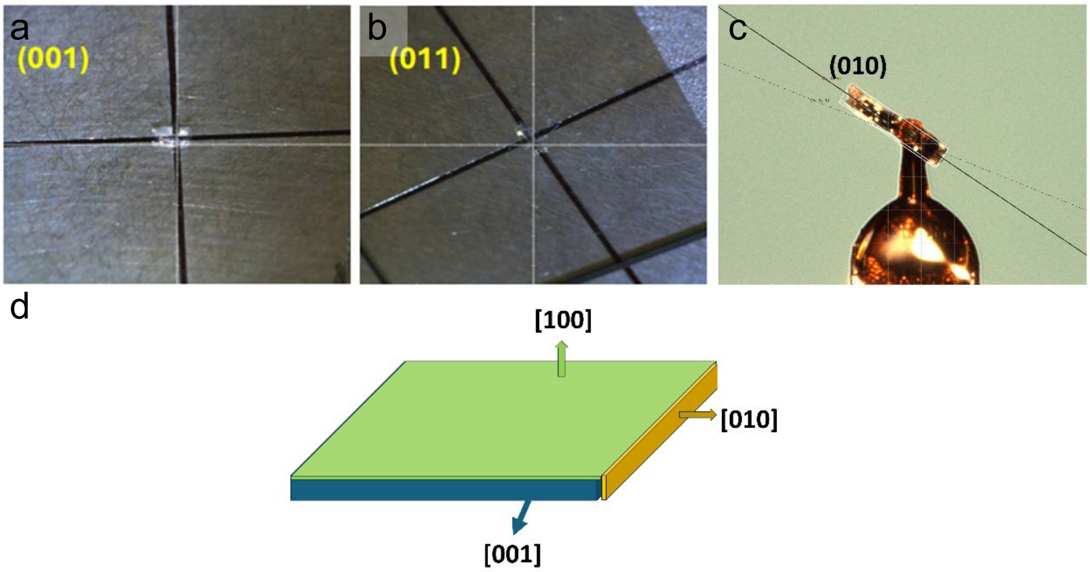

Figure S12. Identification of the crystal faces by X-ray diffraction. (a) and (b) Orientation with a certain plane parallel to the horizontal line as deduced from an azimuthal scan with in-plane 2ϑ angle set to a specified plane. (c) The phase indexing of the single crystal sheet. (d) Schematic description of the crystallographic orientations of the 2D sheets.

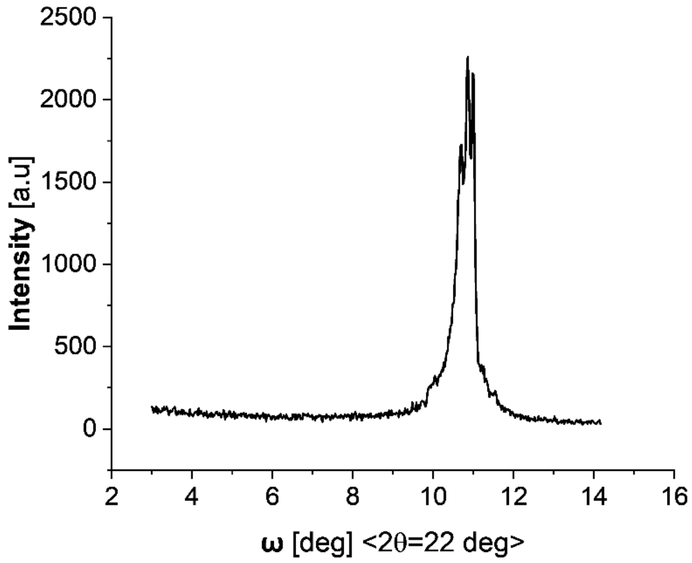

Figure S13. Characterization of the defect density of a theophylline crystal. Rocking curve, ω scan, of (040) peak that was performed on the planar crystals, showing the planar distribution of less than 1° degrees

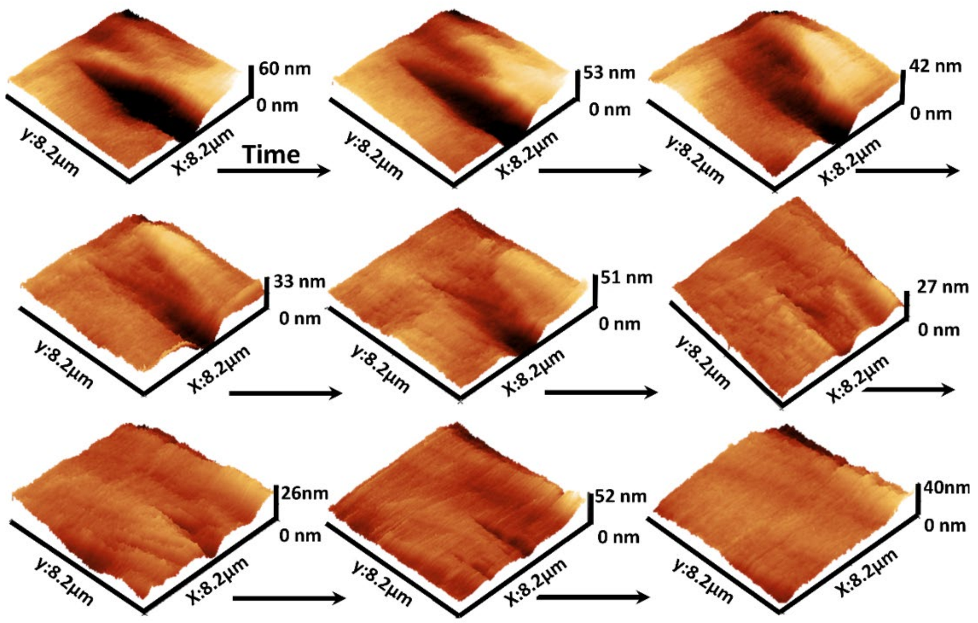

Figure S14. Gradual healing of the crystal by applying a growth solution. 3D AFM images. Theophylline concentration 0.018M. Groove etched along [001] direction.

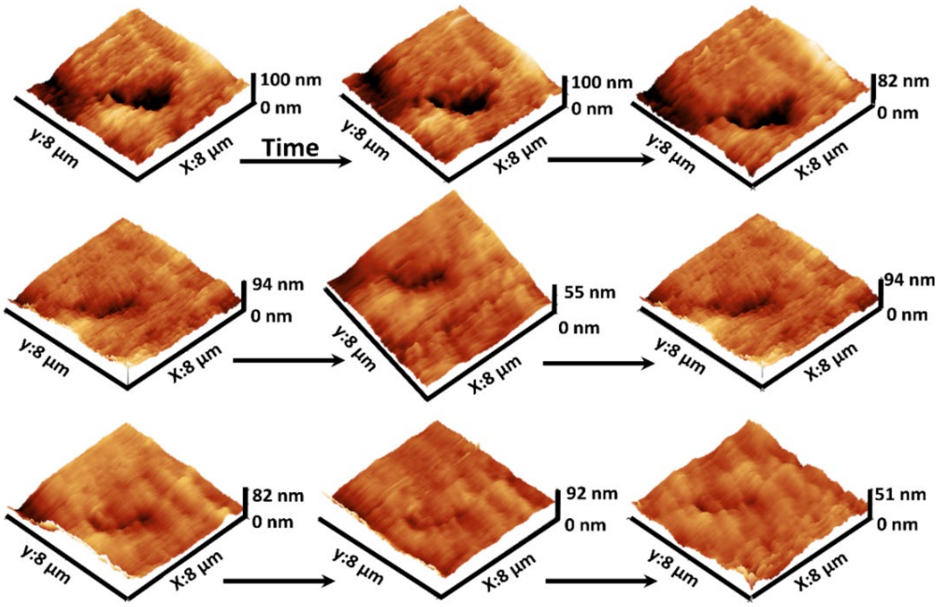

Figure S15. Gradual healing of the crystal by applying a growth solution. 3D AFM images. Theophylline concentration 0.018M. Groove etched along [010] direction.

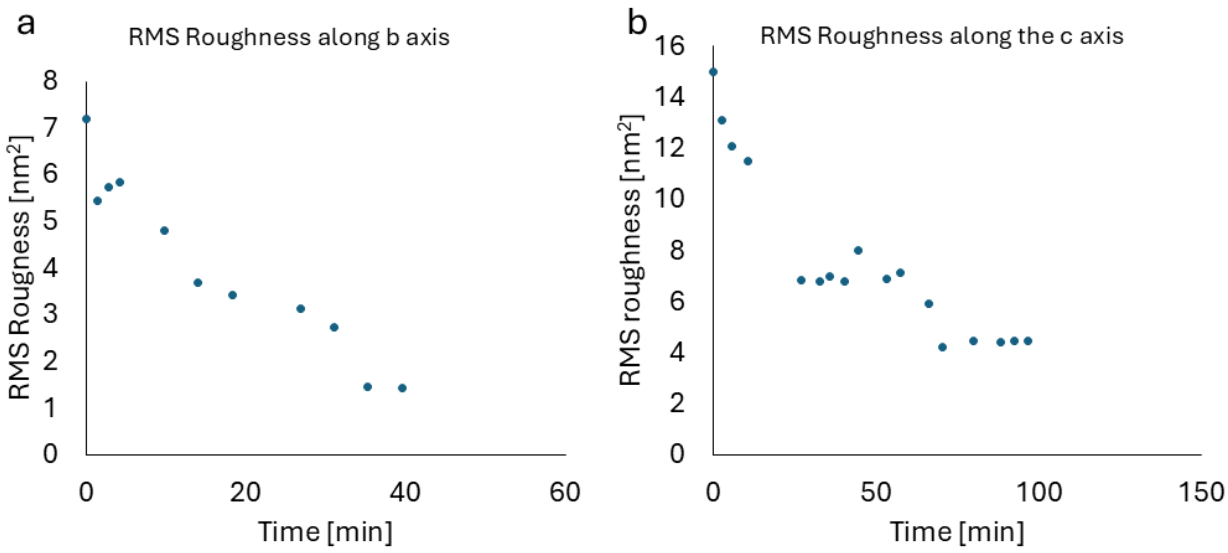

Figure S16. Evolution of the local surface roughness of the crystals during the healing. (a) healing defect along the **b** axis. (b) healing defect along the **c** axis.

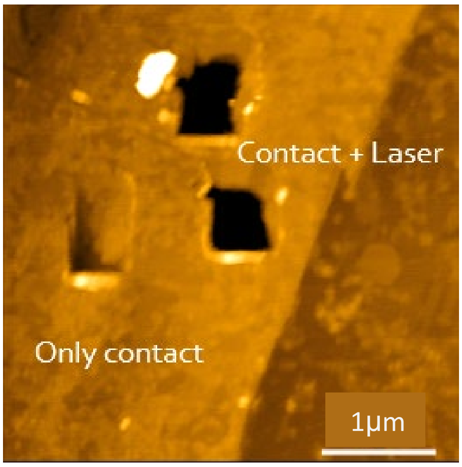

Figure S17. AFM topography of theophylline crystals after scanning lithography. The results of direct thermal scanning lithography vs direct lithography using AFM tip (Fastscan B) without thermal excitation.
